# Supplementary material for: Dynamic transcriptomic profiles of zebrafish gills in response to zinc supplementation
Source: BMC Genomics. 2010 Oct 11;11:553. doi: 10.1186/1471-2164-11-553 (PMC3091702; doi:10.1186/1471-2164-11-553)
Supplement: Additional file 2 — Interactive Direct Interaction Network representing the molecular interactions between zinc, copper, iron, calcium and proteins encoded by transcripts changed by zinc supplementation. Mini web-site containing index.html and hyperlinked pages in subdirectory describing a Direct Interaction Network automatically generated based on curated interactions contained within the proprietary PathwayArchitect database. Ovals represent proteins and the circles symbolize metal ions. Objects are coloured by their abundance in zebrafish at the time-point they were significantly different from the control is a scale from -4 fold (dark green) to +4 fold (dark red). Where significant differences were found at more than one time-point, the colour overlay shows expression at the first instance. Dark blue squares denote 'binding', and light blue squares 'expression'; green squares stand for 'regulation', green diamonds for 'metabolism', and green circles for 'promoter binding'. Arrow heads indicate directionality of the interaction where annotated. All nodes and edges can be further interrogated by selecting the relative area of the image. [file 1471-2164-11-553-S2.zip › PathwayArchitect Zn xs DIN/96204.html]

# PROTEIN: EDNRB

|  |  |
| --- | --- |
| Name | EDNRB |
| Type | PROTEIN |
| Description | endothelin receptor type B |
| Note | Endothelin receptor type B is a G protein-coupled receptor which activates a phosphatidylinoitol-calcium second messenger system. Its ligand, endothelin, consists of a family of three potent vasoactive peptides: ET1, ET2, and ET3. Studies suggest that the multigenic disorder, Hirschsprung disease type 2, is due to mutation in endothelin receptor type B gene. A splice variant, named SVR, has been described; the sequence of the ETB-SVR receptor is identical to ETRB except for the intracellular C-terminal domain. While both splice variants bind ET1, they exhibit different responses upon binding which suggests that they may be functionally distinct. |
| Alias | endothelin-B receptor |
|  | ET-B |
|  | ABCDS |
|  | Hirschsprung disease 2 |
|  | Sox10m1 |
|  | HSCR2 |
|  | ET>B< |
|  | EDNRB |
|  | AU022549 |
|  | Ednrb |
|  | ETRB |
|  | Etb |
|  | ETb |
|  | HSCR |
|  | piebald |
|  | s |
|  | Endothelin receptor Non- selective type |
|  | Ednra |
|  | ETB |


---

|  |  |
| --- | --- |
| GO Component | extracellular space |
|  | integral to membrane |
|  | plasma membrane |
|  | integral to plasma membrane |
|  | membrane fraction |


---

|  |  |
| --- | --- |
| GO ID | GO:0007399 |
|  | GO:0001584 |
|  | GO:0008217 |
|  | GO:0007200 |
|  | GO:0005615 |
|  | GO:0016477 |
|  | GO:0006885 |
|  | GO:0004962 |
|  | GO:0004930 |
|  | GO:0004983 |
|  | GO:0048066 |
|  | GO:0005515 |
|  | GO:0005887 |
|  | GO:0007194 |
|  | GO:0016021 |
|  | GO:0005624 |
|  | GO:0001600 |
|  | GO:0004871 |
|  | GO:0005886 |
|  | GO:0030318 |
|  | GO:0007186 |
|  | GO:0007165 |
|  | GO:0001755 |
|  | GO:0004872 |
|  | GO:0007497 |
|  | GO:0007605 |
|  | GO:0007204 |
|  | GO:0007422 |


---

|  |  |
| --- | --- |
| MIM | MIM:131244 |
|  | MIM:277580 |
|  | MIM:600155 |
|  | MIM:600501 |


---

|  |  |
| --- | --- |
| Connectivity | 275 |


---

|  |  |
| --- | --- |
| Entrez ID | 1910 |
|  | 50672 |
|  | 13618 |


---

|  |  |
| --- | --- |
| Agilent ID | A\_23\_P2831 |
|  | A\_14\_P139074 |
|  | A\_53\_P103522 |
|  | A\_14\_P113603 |
|  | A\_24\_P330263 |
|  | A\_53\_P135852 |
|  | A\_44\_P435618 |
|  | A\_53\_P151926 |
|  | A\_52\_P577662 |
|  | A\_14\_P138768 |
|  | A\_51\_P262964 |
|  | A\_14\_P118701 |
|  | A\_14\_P103903 |
|  | A\_42\_P524460 |
|  | A\_53\_P135839 |
|  | A\_14\_P118485 |


---

|  |  |
| --- | --- |
| Cellular Localization | Plasma membrane |
|  | Membrane |
|  | Extracellular region |
|  | Cell |


---

|  |  |
| --- | --- |
| DbXref | KEGG pathway##04080##Neuroactive ligand-receptor interaction##http://www.genome.jp/dbget-bin/show\_pathway?rno04080+50672 |
|  | KEGG pathway##04080##Neuroactive ligand-receptor interaction##http://www.genome.jp/dbget-bin/show\_pathway?mmu04080+13618 |
|  | KEGG pathway##04020##Calcium signaling pathway##http://www.genome.jp/dbget-bin/show\_pathway?rno04020+50672 |
|  | KEGG pathway##04080##Neuroactive ligand-receptor interaction##http://www.genome.jp/dbget-bin/show\_pathway?hsa04080+1910 |
|  | KEGG pathway##04020##Calcium signaling pathway##http://www.genome.jp/dbget-bin/show\_pathway?mmu04020+13618 |
|  | KEGG pathway##04020##Calcium signaling pathway##http://www.genome.jp/dbget-bin/show\_pathway?hsa04020+1910 |


---

|  |  |
| --- | --- |
| Pathway | Zn xs inventory |
|  | Zn xs DIN |


---

|  |  |
| --- | --- |
| GO Process | elevation of cytoplasmic calcium ion concentration |
|  | G-protein coupled receptor protein signaling pathway |
|  | cell migration |
|  | negative regulation of adenylate cyclase activity |
|  | neural crest cell migration |
|  | peripheral nervous system development |
|  | perception of sound |
|  | signal transduction |
|  | neurogenesis |
|  | pigmentation during development |
|  | regulation of blood pressure |
|  | regulation of pH |
|  | posterior midgut development |
|  | melanocyte differentiation |
|  | G-protein signaling, coupled to IP3 second messenger (phospholipase C activating) |


---

|  |  |
| --- | --- |
| UniGene | Mm.229532 |
|  | Rn.11412 |
|  | Hs.82002 |


---

|  |  |
| --- | --- |
| Affymetrix Probeset ID | 103550\_at |
|  | 1198\_at |
|  | 1387146\_a\_at |
|  | 1423594\_a\_at |
|  | 1426314\_at |
|  | 1437347\_at |
|  | 1459506\_at |
|  | 163124\_s\_at |
|  | 204271\_s\_at |
|  | 204273\_at |
|  | 206701\_x\_at |
|  | 80173\_at |
|  | D13168\_at |
|  | g4503466\_3p\_a\_at |
|  | g4557546\_3p\_at |
|  | rc\_AA818970\_s\_at |
|  | S65355\_at |
|  | S65355\_g\_at |
|  | U32329\_s\_at |
|  | X57764\_s\_at |
|  | RC\_H97587\_s\_at |
|  | RC\_H99393\_s\_at |
|  | TC36230\_at |


---

|  |  |
| --- | --- |
| GO Function | protein binding |
|  | endothelin-B receptor activity |
|  | rhodopsin-like receptor activity |
|  | neuropeptide Y receptor activity |
|  | G-protein coupled receptor activity |
|  | receptor activity |
|  | signal transducer activity |
|  | endothelin receptor activity |


---

|  |  |
| --- | --- |
| Nucleotide | AK085165 |
|  | AK199045 |
|  | AK197551 |
|  | NM\_003991 |
|  | AK205400 |
|  | AK198545 |
|  | AK192804 |
|  | NM\_007904 |
|  | D13168 |
|  | AK202909 |
|  | BC026553 |
|  | AK219164 |
|  | AK201319 |
|  | AK076426 |
|  | AK195149 |
|  | AK198133 |
|  | AK202614 |
|  | AK211542 |
|  | AK201511 |
|  | AK085532 |
|  | AY275463 |
|  | AF074963 |
|  | U32329 |
|  | AY547312 |
|  | NM\_000115 |
|  | X99250 |
|  | AB209198 |
|  | AK218093 |
|  | AK203096 |
|  | AK215891 |
|  | AJ458189 |
|  | S57283 |
|  | AK204434 |
|  | AK196408 |
|  | AF114165 |
|  | S44866 |
|  | AJ458190 |
|  | AK202128 |
|  | AK199342 |
|  | AJ458191 |
|  | AK198739 |
|  | AK206106 |
|  | S65355 |
|  | AK198684 |
|  | AK213746 |
|  | AK211790 |
|  | AK189874 |
|  | AK194698 |
|  | AL139002 |
|  | AK192059 |
|  | AK082103 |
|  | AK199128 |
|  | AJ458188 |
|  | X57764 |
|  | L06623 |
|  | AK215811 |
|  | AK199447 |
|  | AK083415 |
|  | AK190192 |
|  | AK190427 |
|  | AK194687 |
|  | AK214403 |
|  | AK190249 |
|  | U29681 |
|  | BC014472 |
|  | AK192971 |
|  | AK195461 |
|  | U02094 |
|  | D90402 |
|  | M74921 |
|  | AE014177 |
|  | NM\_017333 |


---

|  |  |
| --- | --- |
| Protein | AAH14472 |
|  | AAS38516 |
|  | BAC38409 |
|  | AAP32295 |
|  | AAB60508 |
|  | AAB24922 |
|  | AAM92568 |
|  | AAD24541 |
|  | BAA14398 |
|  | P24530 |
|  | BAC38908 |
|  | BAD92435 |
|  | AAD40187 |
|  | NP\_031930 |
|  | CAD30646 |
|  | NP\_059029 |
|  | BAC39465 |
|  | CAA67623 |
|  | CAD30645 |
|  | AAB28172 |
|  | NP\_000106 |
|  | NP\_003982 |
|  | P21451 |
|  | CAD30647 |
|  | AAA52342 |
|  | AAB19411 |
|  | CAD30648 |
|  | CAA40916 |
|  | AAH26553 |
|  | BAA02445 |
|  | BAC39379 |
|  | P48302 |
|  | AAB25531 |
|  | AAA58465 |
|  | BAC36337 |
|  | CAH72430 |


---

|  |  |
| --- | --- |
| Organism | Mammal |


---

|  |  |
| --- | --- |
| Location | 14 51.0 cM (Mus musculus) |
|  | chromosome 14, 14 51.0 cM, 14 E2.3 (Mus musculus) |
|  | chromosome 15 (Rattus norvegicus) |
|  | chromosome 13, 13q22 (Homo sapiens) |


---

|  |  |
| --- | --- |
